# Biofunctional Understanding and Judgment of Size

**DOI:** 10.3389/fpsyg.2016.00436

**Published:** 2016-03-24

**Authors:** Zheng Jin, Yang Lee, Zheng Yuan

**Affiliations:** ^1^Zhengzhou Normal University Zhengzhou, China; ^2^University of California, Davis Davis, CA, USA; ^3^Department of Psychology, Gyeongsang National University Jinju, South Korea; ^4^Haskins Laboratories, Yale University New Heaven, CT, USA

**Keywords:** embodied cognition, biofunctional understanding, action, affordances, size judgments

## Abstract

Research has shown that the meaningfulness of the material increases judged size, whereas symmetry decreases size judgments. These findings have been interpreted in terms of information processing, with a greater quantity of information leading to a judgment of larger size. An alternative view based on biofunctional understanding theory emphasizes the quality of affordance-triggered biological activity as reported and observed in attitudes toward playing sports, effortless understanding, knowledge-in-action, meditative wisdom, and body–mind cycle of adaptation. This alternative implies that affordance biofunctional activity is naturally size-diminishinging as it moves toward coherence and size-expanding as it moves away from coherence influencing judgments of size accordingly. Here we tested this hypothesis in the realm of sensorimotor integration. Our first experiment showed that phonologically unpronounced or symmetric symbols elicit smaller size judgments than phonologically pronounced and asymmetric symbols. Next, we manipulated the quantity of meaning with the affordance (possibilities for biofunctional activity) orthogonally in a second experiment; results indicated that meaning affects size judgments only in the absence of phonological information. We conclude that the biofunctional activity affordance may be responsible for observed differences in size judgment.

## Introduction

Psychonomic studies have shown that processing by the visual system may allow a direct translation of physical size into mental size (Stevens and Galanter, 1957; Casasanto and Boroditsky, 2008). Although, size judgments appear to accurately reflect actual size, they are not always unbiased. For example, coins are estimated to be larger by children from relatively poor economic backgrounds than by children from more aﬄuent economic backgrounds (Bruner and Goodman, 1947). The best-known example of this phenomenon, called the Ebbinghaus illusion, illustrates that in some circumstances, size judgments are context-dependent (Coren and Miller, 1974; Coren and Enns, 1993; Roberts et al., 2005). Studies on word and non-word size judgments have demonstrated a word-superiority effect (e.g., Reber et al., 2004; also see New et al., 2016), i.e., words are judged as being larger than non-words, providing evidence that context manipulations bias size judgments.

At the very beginning, the contextual effects for size judgment were interpreted as an effect of processing fluency (Reber et al., 2004), which is a measure of how much information can be processed per time unit. According to the concept of processing fluency, a word is processed more easily when more information is available that allows the generation of concept-driven hypotheses about the word; therefore, processing more information per unit time may result in the translation of physical size into a larger mental size. Symmetric forms would thus be judged to be larger than asymmetric forms, given the fact that symmetric shapes are more easily processed than asymmetric shapes due to reduced visual complexity (Royer, 1981; Wurtz et al., 2008). However, contrary to this expectation, recent studies have shown that symmetry decreases size judgment, whereas forms and shapes that convey meaning are judged as larger (e.g., Reber et al., 2010, 2014). These results refuted the processing fluency theory but proposed an information-based mechanism, in which judgments on size depend on how much information must be processed. Specifically, less information is processed for less visually complex objects (e.g., symmetric shapes) because the information is redundant or simply reduced, thus saving on cognitive resources and translating into a smaller mental size. On the other hand, additional semantic information integrated into meaningful material (e.g., a word) would translate into a larger mental size.

Another explanation for size judgments uses the concept of affordance and the lens of biofunctional understanding theory (Iran-Nejad, 1987a,b, 2013) that emphasizes the quality of affordance-triggered biological activity (Jin et al., 2015) as reported and observed in attitudes toward playing sports (Zengaro et al., in press), effortless understanding (Auble et al., 1979), knowledge-in-action (Schön, 1983), biofunctional interest (Iran-Nejad, 1987a), meditative wisdom (Rosch, 2000), and body–mind cycle of reflection (Iran-Nejad and Gregg, 2001). This alternative has been endorsed by ecological perspectives, which have long appreciated that vision is inextricably linked to the control of action (Gibson, 1979; Fajen, 2007) and implies that affordance-trigered biofunctional activity contracts size judgments as it moves toward coherence and expands size judgments as it moves away from coherence.

In affordance, the manner in which one uses his or her body to interact with the environment affects his or her perception of the environment. In other words, the object is perceived in terms of the possibility for action—so called “perception through action” (Turvey and Kugler, 1984; Witt and Proffitt, 2005). Based on the assumption that judgments of physical size are good proxies for perceived size, a growing number of studies have found that individuals perceive objects not only through bodily actions but also by controlling affordance before the action (e.g., Jin and Lee, 2013). For example, a creek affords the action of jumping and vision changes with the progress of the action. Before jumping, a person may perceive the narrowest section afforded by the creek for jumping. In natural cognitive systems, increased sensory complexity, along with the machinery used to interpret such complexity, is generally associated with an increasing ability to interact with and manipulate the environment, which is facilitated by increasing motor capabilities (see Milner and Goodale, 1995). People may perceive visually complex objects as larger in response to the more salient, actionable properties (i.e., affordance, see also Lee et al., 2012).

By the same logic of action-specific perception, people would also translate words into a larger size than non-words through affordance. As proposed by articulation transformational phonology (Chomsky and Halle, 1968), the phonological process is based on articulatory gestures, which are the actions necessary to enunciate the language. Examples of gestures are the mouth movements of speech or the hand movements of sign language. This suggests that articulation can process scripts, thereby transforming the inner code of language (e.g., Browman and Goldstein, 1986, 1992, 1995). Both words and non-words can be regarded as a combination of discrete, physical signs (e.g., letters); however, for example, the articulatory phonology involved in c-u-p is more easily to afford the articulator (e.g., the lips, tongue, glottis, velum) movements than a figure of a cup. Accordingly, words are predicted to have a different perceived size than non-words because of the greater effort with which the phonological process allows speech production. Indeed, it has been argued that the speech signal should be viewed as an embodied, intentional act, and placed in the context of a wide range of interaction affordances (e.g., Worgan and Moore, 2010).

Both the amount of processed information and affordance-based biofunctional understanding appear appropriate for explaining how visual complexity and lexical knowledge translate into estimates of size. However, the amount of processed information has been neither properly defined nor quantified directly but only inferred from the characteristics of the materials in previous research (Reber et al., 2014). Thus, it is possible that the judgment of larger size for meaningful materials is attributable to the extent to which phonological information is contained in the quantity of information. Hence, it is not possible to assume that the mechanism underlying size judgments is only based on the quantity of processed information. It is also plausible that size judgments depend on the possibility of (articulatory) action that is afforded by phonology. However, because the phonological level or effort has never been quantified for correlation with size judgments, the mechanism underlying size judgments is still vague.

In the present study, we first conducted an experiment to examine the independent contribution of phonological information to producing size judgments. In a second experiment, we then investigated the information-based and affordance-based mechanisms through which size judgments are explained, by manipulating the amount of processed information and the possibility for action orthogonally within this experiment.

## Materials and Methods

### Experiment 1

#### Participants

Thirty nine undergraduate students from three Korean Language School at Zhengzhou City (17 females, 22 males) participated in the experiment for payment. Mean age was *M* = 22.59 (*SD* = 1.17) years. They were native Chinese speakers and 38 had passed the Test of Proficiency in Korean (TOPIK, Level 4). Note that the design applies to both native and non-native Korean speakers in theory. For this study, however, especially, the second experiment, sufficient knowledge about Chinese language is required due to the need of implicit semantic/phonological processing. The study was approved of by the Academic Ethics Committee of the Zhengzhou Normal University. Informed consent was obtained from each participant before the testing.

#### Materials

**FIGURE 1:**
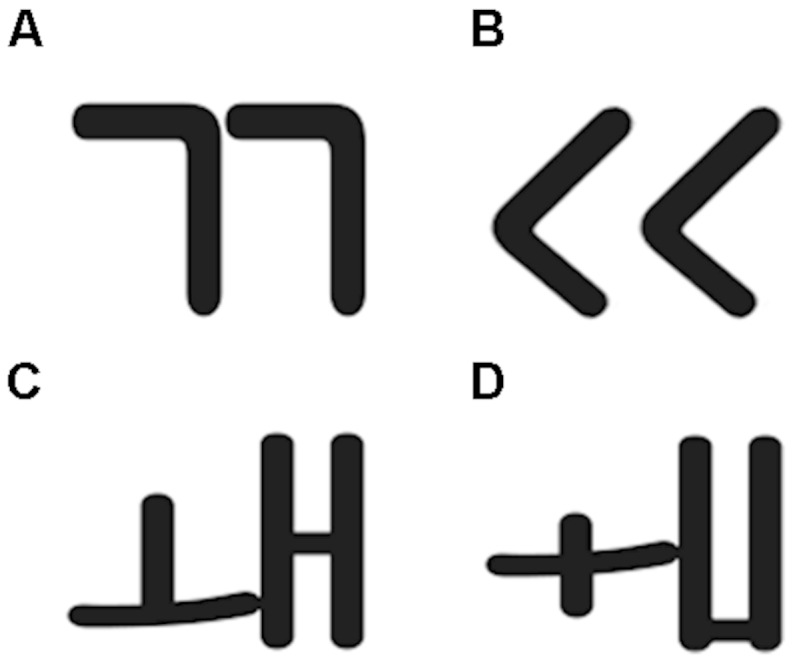
**(Top) A vowel **(A)** and an unpronounced symbol **(B)** that were assembled from this vowel; (Bottom) A consonant **(C)** and an unpronounced symbol **(D)** that were assembled from this consonant**.

A total of six letters were chosen from the Korean language system, with three in each of two symmetries. The symmetric letters were three vowels: 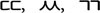 (P+S+, where *P* represents phonological information and *S* represents symmetry, **Figure 1A**), and the asymmetric letters were three consonants: 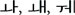 (P+S-; **Figure 1C**). In both cases, the designation “plus” (+) is used when the production constraint is respected within the orthography; the designation “minus” (-) is used when the production constraint is absent from the orthography. In addition, we constructed unpronounceable symbols that were assembled from parts of the Korean letters (P-S+ versus P-S-; **Figure 1B** versus **Figure 1D**). All of the symmetric items possessed translatory symmetry, which is the repetition of elements without changing its vertical position (c.f., Reber et al., 2014).

Each stimulus was drawn in four font sizes (34, 36, 38, or 40 mm), with width and height held constant (as in Reber et al., 2014). This yielded 48 experimental trials: 3 (symbol) × 2 (pronunciation) × 2 (symmetry) × 4 (font size). The mean number of strokes (i.e., visual features, such as horizontal, vertical, and diagonal lines) was the same among the four cells (pronunciation by symmetry), that is, 4.67. This indicated that the participants processed the same level of visual complexity.

#### Procedure

The participants were seated in front of a computer monitor and given information about the purpose of the experiment. They were told that we are studying visual judgment and were asked to give their consent to participate in the experiment by pressing a key on the keyboard. The participants were then instructed to rate the size of the numbers on a scale from 1 (small) to 9 (large), using the number keys on the keyboard.

The participants were first presented with a black fixation cross on a white background for 500 ms to focus their attention on the center of the screen. Then, a stimulus, symmetric or asymmetric and consisting of Korean letters or symbols, was presented for 200 ms in the center of the screen. The participants had to judge how large the target item was on the nine-point scale. The order of the stimuli was random.

After the size judgment task, the participants were required to rate the meaning of each stimulus on a seven-point scale. The instruction given was “Please indicate to what extent the stimulus is meaningful to you.” Larger values indicated that a stimulus was more meaningful.

#### Results and Discussion

**FIGURE 2:**
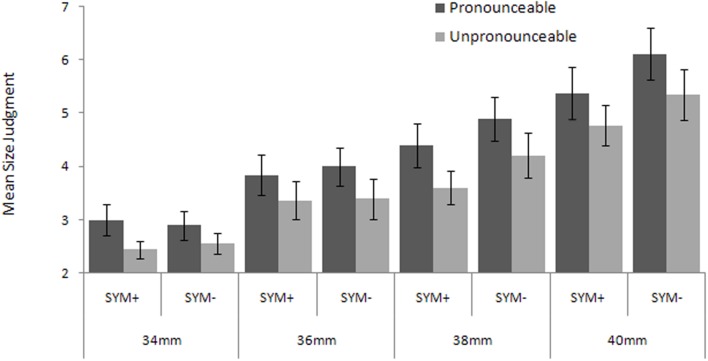
**Mean size judgment as a function of pronunciation, physical font size, and symmetry (SYM+ versus SYM-).** Error bars indicate 95% confidence bounds (O’Brien and Cousineau, 2014).

The mean rating for each of the pronunciation × symmetry × font size conditions for each participant was computed (**Figure 2**). The alpha level was set at 0.05 for all analyses.

The 2 × 2 × 4 ANOVA within subjects ANOVA, with all variables, pronunciation (letters versus unpronounceable symbols), symmetry (symmetric versus asymmetric), and font size (34, 36, 38, 40 mm) revealed an effect of pronunciation, *F*(1,37) = 35.45, *p* < 0.001, $\eta_{p}^{2}$ = 0.49, indicating that the size was judged larger with letters (*M* = 4.31, *SD* = 1.58) than with unpronounceable symbols (*M* = 3.71, *SD* = 1.42). There was also a strong effect of physical font size, *F*(3,111) = 98.59, *p* < 0.001, $\eta_{p}^{2}$ = 0.73. Not surprisingly, larger font sizes were judged to be larger. *Post hoc* multiple comparisons showed significant differences between all font sizes: 34 mm (*M* = 2.72, *SD* = 0.07); 36 mm (*M* = 3.65, *SD* = 0.09), 38 mm (*M* = 4.28, *SD* = 0.11), and 40 mm (*M* = 5.40, *SD* = 0.16). The effect of symmetry on size judgments that was observed in previous studies was replicated here, *F*(1,37) = 15.98, *p* < 0.001, $\eta_{p}^{2}$ = 0.32. The participants judged symmetric stimuli (*M* = 3.85, *SD* = 0.07) as being smaller than asymmetric stimuli (*M* = 4.17, *SD* = 0.07). However, there was a significant interaction between symmetry and size, *F*(3,111) = 4.20, *p* < 0.05, $\eta_{p}^{2}$ = 0.10. To evaluate this interaction, a simple contrast was performed between symmetric and asymmetric patterns for each size. The interaction derives from an association between larger physical size and greater symmetry (*p* < 0.01, $\eta_{p}^{2}$ > 0.24). The difference between symmetric and asymmetric patterns was significant only for the larger font sizes: for 38 mm, *M_s+_* = 4.00, *SE* = 0.13; *M_s-_* = 4.55, *SE* = 0.15, and for 40 mm, *M_s+_* = 5.07, *SE* = 0.16; *M_s-_* = 5.72, *SE* = 0.21.

In the Korean language system, neither these vowels nor these consonants possess meaning; they only hold meaning when they comprise an entire syllable (Taylor, 1980). However, any symbol may have meaning even if it exists in a non-linguistic form, such as “**×**” (a cross) or the unpronounceable symbols used in this study. Our analyses showed that the spreading meaning activation was well-controlled, *F*(3,120) = 1.99, *p* = 0.12. This result suggests that judged size is increased when phonological information is available. The conclusion that pronounced properties increased font size appears to be consistent with the information-based concept, which assumes that a greater quantity of information increases the judgments of font size.

However, note that, according to the information-based account, additional phonological information should perform the same function as semantic information that can be translated into a larger mental size. Thus, size judgments should differ in manipulations that affect the magnitude of either semantic information or phonological information. In contrast, according to the affordance-based account, estimated proximal size is scaled intrinsically according to the current level of visually perceived articulatory form (c.f., Lee et al., 2012); hence, only the manipulation that affects the phonological level should affect size judgments. Our second experiment was designed to test which account underlies size judgments.

### Experiment 2

#### Participants

Thirty-two Chinese undergraduate students who were majoring (or studying) in Korean language and had passed the Test of Proficiency in Korean (TOPIK, Level 4) participated in the rating study. Thirty-six students from the same population (19 females, 17 males) participated in the main experiment for payment. The mean age was 22.42 (*SD* = 1.48) years. None of the participants had taken part in the first experiment.

#### Materials

A set of disyllabic Korean words consisted of Sino-Korean words correspond closely to modern Chinese (Mandarin) in phonological structure and *pure* Korean words lacking a clear Chinese phonological translation were selected from a corpus of Korean words that was developed by the Korean Advanced Institute of Science and Technology (KAIST, 1999). A different set of Chinese words, possessed similar word frequency to the Chinese words from which the Sino-Korean words were derived, was chosen for generating Korean disyllabic non-words. Chinese word frequency was estimated from a database with a corpus of over 973,338 Chinese dissyllable words (Bigram frequencies and mutual information in Modern Chinese, Da, 2004). Given the fact that each Korean syllable possesses one-to-one correspondence between letters and phonemes (Taylor, 1980), a number of Korean disyllables were then created to resemble the pronunciations of these Chinese words and none of them are words.

The 32 participants (see Participants) rated the sets of disyllabic Korean words from 1 (not very) to 7 (very) in terms of familiarity and rated all disyllables (both words and non-words) in terms of phonological similarity to Chinese. Thirty-two disyllables were finally chosen, with eight in each of four word (word versus non-word, i.e., W+ versus W-) × phonological similarity to Chinese (high versus low, i.e., C+ versus C-) cells. The mean rating of familiarity did not differ in phonological similarity (*M* = 4.56, *SD* = 1.09). In addition, the average frequency counts for the Chinese words which served as origin of the Sino-Korean words and the Chinese words served as basis for creating Korean syllables were 579 (±355) and 575 (±357), respectively. The mean rating of phonological similarity for the eight Sino-Korean words was 5.16 ± 0.66 and for the eight *pure* Korean words was 1.79 ± 0.50, and for non-words created from Chinese words was 5.41 ± 0.55, and for non-words created from *pure* Korean was 1.6 ± 0.22, *ps >* 0.05. The number of strokes was equal among the four cells (*F* < 1; See Appendix for stimuli List).

#### Procedure

The procedure was the same as in Experiment 1, with the exception that each stimulus was drawn with only one size; the stimulus width and height were 37 mm. In this experiment, the same instruction as the first experiment was adopted, that is, participants were ostensibly told that the stimuli would be presented with size variation.

#### Results and Discussion

**FIGURE 3:**
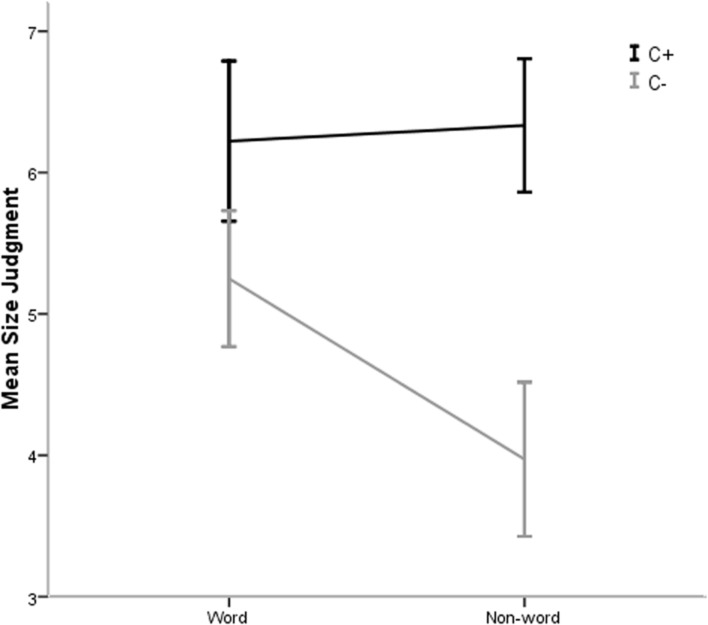
**Mean size judgment as a function of phonological similarity to Chinese and word (Phonological similarity to Chinese is represented by “C+” and that without this phonological similarity is represented by “C-”).** Error bars indicate 95% confidence bounds (O’Brien and Cousineau, 2014).

An analysis of variance (ANOVA) on word/non-word (W+ versus W-) and phonological similarity to Chinese (C+ versus C-) revealed a marginally significant effect of word (*M_w+_* = 5.74, *SD* = 1.60, and *M_w_*_-_ = 5.15, *SD* = 1.91); *F*(1,35) = 3.70, *p* = 0.06, $\eta_{p}^{2}$ = 0.10; a significant effect of phonological similarity (*M_c+_* = 6.28, *SD* = 1.53 and *M_c_*_-_ = 4.61, *SD* = 1.64), *F*(1,35) = 59.32, *p* < 0.001, $\eta_{p}^{2}$ = 0.63; and a significant word by phonological similarity interaction, *F*(1,35) = 7.18, *p* < 0.05, $\eta_{p}^{2}$ = 0.17. Simple effect tests were performed for each level of phonological similarity to Chinese. When the similarity to Chinese phonology was absent, there was an effect of word, *F*(1,35) = 10.44, *p* < 0.005, $\eta_{p}^{2}$ = 0.29. *Post hoc* multiple comparisons indicated that the size judgments were larger with words (*M* = 5.25, *SE* = 0.24) than with non-words (*M* = 3.97, *SE* = 0.27). However, when Chinese phonology potentially existed, there was no significant effect of word ($\eta_{p}^{2}$ < 0.01). Accordingly, the interaction arises because meaning affects the size estimate only when there is a lack of phonological information (**Figure 3**). The outcome of Experiment 2 supports the hypothesis that size judgment was affected by the perceived phonological level.

## Discussion

Although, judged size corresponds well with real size, context manipulations can bias size judgments, as shown by Bruner and Goodman (1947) and by the Ebbinghaus illusion (e.g., Coren and Miller, 1974). The recent study by Reber et al. (2014) in which asymmetric characters were judged to be larger than symmetric characters and meaningful numbers were judged to be larger than meaningless characters derived from number stimuli, suggests that the quantity of processed information influences size judgments. We replicated this finding in Experiment 1: asymmetric symbols were judged to be larger than symmetric symbols. In addition, we showed that pronounced letters were judged to be larger than unpronounced symbols by manipulating symmetry and pronunciation orthogonally. These results appear consistent with the information-based mechanism for size judgments, i.e., size judgments depended on how much phonological information the participants processed. However, it raises the possibility that affordance, i.e., the possibility for biofunctional action rendered by the symbols, rather than the quantity of information processed, drives the effect on size judgments.

It is clear that the complexity of interaction that a system can demonstrate, i.e., its motor capabilities, is determined to some extent by the complexity of its perceptual system. It is perhaps less clear that the complexity of the perceptual system is determined by the complexity of the motor capabilities. However, this cyclical causality linking perceptual and motor capabilities is supported by a large body of research in the modern cognitive sciences and has firm philosophical (Lakoff and Johnson, 1999), neurophysiological foundations (Garbarini and Adenzato, 2004). In particular, in the theory of embodiment, the term affordance has been used to state that the world can be perceived not only in terms of object shapes and spatial relationships but also in terms of the possibilities for action (Gibson, 1977). Asymmetry enacts more capacity to differentiate in which effortful mental reflection is demanded. By contrast, symmetry is an example of biofunctional integration, as biofunctional understanding is immediate and effortless integration, and thus was judged as having smaller size.

However, before rejecting an information-based explanation, we needed to examine whether manipulations of the phonological level could account for the size judgments independent of the manipulations of other processed information. Our second experiment showed an interaction effect between phonological and semantic information on size judgment. Obviously, Korean loanwords from Chinese (W+C+) possess more (additional semantic) information than non-words that have phonological translation from Chinese (W-C+), but they were not judged to be larger. It is thus unlikely that the mechanism underlying size judgments was based on the quantity of information processed. Instead, any manipulation of perceived phonological level, rather than the semantic level (meaning), would influence size judgments. Theories appealing to information content are strongly dismissed in this study.

The result that processing phonological information can enlarge the size estimation independent of processing semantic information is consistent with the idea of biofunctional embodiment, which proposes that actions are the basis for physical judgments and probably underlie other basic cognitive interpretations of sensory stimuli (Jin et al., 2015) — no matter what the action is — it may be either actions that demand for differentiation (e.g., brain activity) or actions with effort (e.g., pronouncing). According to biofunctional understanding, lack of these possibilities may facilitate immediate and effortless integration by means of inherent downsizing processes. The context that affords an action (e.g., an articulator movement) enables a person to perceive the form that is propitious to the action. To further illustrate this point, consider the prior findings that compared with non-words, words provide more possibilities for action by vocal organs and thus are perceived as having a larger physical size (Reber et al., 2004). Similarly, numbers were judged as having a larger size than meaningless characters (Reber et al., 2014). The finding that W+C- was estimated to be larger than W-C- in this study might be explained by the context of meaningful materials allowing greater articulation.

### Limitation

Rating-scale judgments of size might be considered to be less psychophysically metric, and limited to get effects on perceived (rather than judged size) which satisfies readers in perception science. The physical sizes of all the stimuli were identical in the second experiment, indeed, to some degree, leaving participants forced to make distinctions on a rating scale based on whatever they could in the absence of any real size differences. Future studies using forced choice comparisons are needed to evidence the current conclusion in a more compelling way.

The objective entity may or may not have sufficient information for articulator movements; however, it has been well-documented that people have an affordance-control ability to perceive “actableness” in favor of the action (e.g., Jin et al., 2015). Although our study does not supplement evidence for this ability, it may stimulate studies that demonstrate whether people perceive the objective world in terms of affordance-control abilities, which are instruments for perception and production of language.

## Author Contributions

ZJ conceived the study, designed the trial and drafted the manuscript, ZY supervised the data collection, and YL, ZY contributed to the revision.

## Conflict of Interest Statement

The authors declare that the research was conducted in the absence of any commercial or financial relationships that could be construed as a potential conflict of interest. The reviewer FS and handling Editor declared their shared affiliation, and the handling Editor states that the process nevertheless met the standards of a fair and objective review.

## References

[B1] Auble P. M. Franks J. J. Soraci S. A. (1979). Effort toward comprehension: elaboration or “aha!”? *Mem. Cogn.* 7 426–434. 10.3758/BF03198259

[B2] Browman C. P. Goldstein L. (1986). Towards an articulatory phonology. *Phonol. Yearbook* 3 219–252. 10.1017/S0952675700000658

[B3] Browman C. P. Goldstein L. (1992). Articulatory phonology: an overview. *Phonetica* 49 155–180. 10.1159/000261913 1488456

[B4] Browman C. P. Goldstein L. (1995). “Dynamics and articulatory phonology,” in *Mind as Motion*, eds Gelder T. van Port R. F. (Cambridge, MA: MIT Press), 175–193.

[B5] Bruner J. S. Goodman C. C. (1947). Value and need as organizing factors in perception. *J. Abnorm. Soc. Psychol.* 42 33–44. 10.1037/h0058484 20285707

[B6] Casasanto D. Boroditsky L. (2008). Time in the mind: using space to think about time. *Cognition* 106 579–593. 10.1016/j.cognition.2007.03.004 17509553

[B7] Chomsky N. Halle M. (1968). *Sound Pattern of English.* New York, NY: Harper & Row.

[B8] Coren S. Enns J. T. (1993). Size contrast as a function of conceptual similarity between test and inducers. *Percept. Psychophys.* 54 579–588. 10.3758/BF03211782 8290327

[B9] Coren S. Miller J. (1974). Size contrast as a function of figural similarity. *Percept. Psychophys.* 16 355–357. 10.3758/BF03203955 8290327

[B10] Da J. (2004). *Bigram Frequencies and Mutual Information in Modern Chinese.* Available at: http://lingua.mtsu.edu/chinese-computing/

[B11] Fajen B. R. (2007). Affordance-based control of visually guided action. *Ecol. Psychol.* 19 383–410. 10.1080/10407410701557877

[B12] Garbarini F. Adenzato M. (2004). At the root of embodied cognition: cognitive science meets neurophysiology. *Brain Cogn.* 56 100–106. 10.1016/j.bandc.2004.06.003 15380880

[B13] Gibson J. J. (1977). *The Theory of Affordances.* Hillsdale, NJ: Lawrence Erlbaum.

[B14] Gibson J. J. (1979). *The Ecological Approach to Visual Perception.* Boston, MA: Houghton-Miﬄin.

[B15] Iran-Nejad A. (1987a). Cognitive and affective causes of interest and liking. *J. Educ. Psychol.* 79 120–130. 10.1037/0022-0663.79.2.120

[B16] Iran-Nejad A. (1987b). “The schema: a long-term memory structure or a transient structural phenomena,” in *Understanding Readers’ Understanding*, eds Tierney R. J. Andres P. L. Mitchell J. N. (Mahwah, NJ: Lawrence Erlbaum), 109–127.

[B17] Iran-Nejad A. (2013). The paradox of the missing biological function in understanding: implications for moral and general education. *Int. J. Educ. Psychol.* 2 1–18.

[B18] Iran-Nejad A. Gregg M. (2001). The brain-mind cycle of reflection. *Teach. Coll. Rec.* 103 868–895. 10.1111/0161-4681.00137

[B19] Jin Z. Lee Y. (2013). Enlargement of perceived target size: intentional or natural? *Percept. Mot. Skills* 117 855–867. 10.2466/24.27.PMS.117x26z0 24665802

[B20] Jin Z. Lee Y. Zhu J. (2015). Control your mind, make affordance available. *Front. Psychol.* 6:96 10.3389/fpsyg.2015.00096 PMC4330679 25741298

[B21] KAIST (1999). *KAIST Corpus.* Available at: http://semanticweb.kaist.ac.kr/home/index.php/KAIST_Corpus

[B22] Lakoff G. Johnson M. (1999). *Philosophy in the Flesh: The Embodied Mind and its Challenge to Western Thought.* New York, NY: Basic Books.

[B23] Lee Y. Lee S. Carello C. Turvey M. T. (2012). An archer’s perceived form scales the “hitableness” of archery targets. *J. Exp. Psychol. Hum. Percept. Perform.* 38 1125–1131. 10.1037/a0029036 22731994

[B24] Milner A. D. Goodale M. A. (1995). *The Visual Brain in Action.* Oxford: Oxford University Press.

[B25] New B. Doré-Mazars K. Cavézian C. Pallier C. Barra J. (2016). The letter height superiority illusion. *Psychon. Bull. Rev.* 23 291–298. 10.3758/s13423-014-0753-8 26370216

[B26] O’Brien F. Cousineau D. (2014). Representing Error bars in within-subject designs in typical software packages. *Quant. Methods Psychol.* 10 56–67. 10.3758/s13428-013-0441-z

[B27] Reber R. Christensen B. T. Meier B. (2014). Effects of meaning and symmetry on judgments of size. *Front. Psychol.* 5:1270 10.3389/fpsyg.2014.01270 PMC4219381 25408681

[B28] Reber R. Wurtz P. Knapstad M. Lervik L. V. (2010). Polarity correspondence in comparative number magnitude judgments. *Psychon. Bull. Rev.* 17 219–223. 10.3758/PBR.17.2.219 20382923

[B29] Reber R. Zimmermann T. D. Wurtz P. (2004). Judgments of duration, figure-ground contrast, and size for words and nonwords. *Percept. Psychophys.* 66 1105–1114. 10.3758/BF03196839 15751469

[B30] Roberts B. Harris M. G. Yates T. A. (2005). The roles of inducer size and distance in the Ebbinghaus illusion (Titchener circles). *Perception* 34 847–856. 10.1068/p5273 16124270

[B31] Rosch E. (2000). The brain between two paradigms: can biofunctionalism join wisdom intuitions to analytic science? *J. Mind Behav.* 21 189–204.

[B32] Royer F. L. (1981). Detection of symmetry. *J. Exp. Psychol. Hum. Percept. Perform.* 7 1186–1210. 6458647 10.1037//0096-1523.7.6.1186

[B33] Schön D. A. (1983). *The Reflective Practitioner: How Professionals Think in Action.* New York, NY: Basic Books.

[B34] Stevens S. S. Galanter E. H. (1957). Ratio scales and category scales for a dozen perceptual continua. *J. Exp. Psychol.* 54 377–411. 10.1037/h0043680 13491766

[B35] Taylor I. (1980). “The Korean writing system: an alphabet? A syllabary? A logography?,” in *Processing of Visible Language*, eds Kolers P. A. Wrolstad M. E. Bouma H. (New York, NY: Plenum), 67–82.

[B36] Turvey M. T. Kugler P. N. (1984). “An ecological approach to perception and action,” in *Human Motor Action: Bernstein Reassessed*, ed.Whiting H. T. A. (Amsterdam: North-Holland), 373–412.

[B37] Witt J. K. Proffitt D. R. (2005). See the ball, hit the ball: apparent ball size is correlated with batting average. *Psychol. Sci.* 16 937–938. 10.1111/j.1467-9280.2005.01640.x 16313656

[B38] Worgan S. F. Moore R. K. (2010). Speech as the perception of affordances. *Ecol. Psychol.* 22 327–343. 10.1080/10407413.2010.517125

[B39] Wurtz P. Reber R. Zimmermann T. D. (2008). The feeling of fluent perception: a single experience from multiple asynchronous sources. *Conscious. Cogn.* 17 171–184. 10.1016/j.concog.2007.07.001 17697788

[B40] Zengaro S. Iran-Nejad A. Schumacher R. Zengaro F. (in press) Understanding adolescent attitudes toward sports aggression: an integrated perspective. *J. Educ. Hum. Dev.*

