# Corrigendum: Biofunctional Understanding and Judgment of Size

**DOI:** 10.3389/fpsyg.2018.01515

**Published:** 2018-08-27

**Authors:** Zheng Jin, Yang Lee, Zheng Yuan

**Affiliations:** ^1^Zhengzhou Normal University, Zhengzhou, China; ^2^University of California, Davis, Davis, CA, United States; ^3^Department of Psychology, Gyeongsang National University, Jinju, South Korea; ^4^Haskins Laboratories, Yale University, New Heaven, CT, United States

**Keywords:** embodied cognition, biofunctional understanding, action, affordances, size judgments

In the original article, there was an error. It was stated that “Chinese word frequency was estimated from a database with a corpus of over 973,338 Chinese dissyllable words (Modern Chinese Character Frequency List, Da, 2004).” The database title was incorrect and may affect the future replication of this research.

A correction has been made to the *Materials and Methods*, sub-section *Experiment 2*, sub sub-section *Materials*, Paragraph 1:

A set of disyllabic Korean words consisted of Sino-Korean words correspond closely to modern Chinese (Mandarin) in phonological structure and *pure* Korean words lacking a clear Chinese phonological translation were selected from a corpus of Korean words that was developed by the Korean Advanced Institute of Science and Technology (KAIST, 1999). A different set of Chinese words, possessed similar word frequency to the Chinese words from which the Sino-Korean words were derived, was chosen for generating Korean disyllabic non-words. Chinese word frequency was estimated from a database with a corpus of over 973,338 Chinese dissyllable words (Bigram frequencies and mutual information in Modern Chinese, Da, 2004). Given the fact that each Korean syllable possesses one-to-one correspondence between letters and phonemes (Taylor, 1980), a number of Korean disyllables were then created to resemble the pronunciations of these Chinese words and none of them are words.

The authors apologize for this error and state that this does not change the scientific conclusions of the article in any way.

The original article has been updated.

## Conflict of interest statement

The authors declare that the research was conducted in the absence of any commercial or financial relationships that could be construed as a potential conflict of interest.
