# Supplementary material for: Biofunctional Understanding and Judgment of Size
Source: Front Psychol. 2016 Mar 24;7:436. doi: 10.3389/fpsyg.2016.00436 (PMC4805645; doi:10.3389/fpsyg.2016.00436)
Supplement: Supplementary file 1 [file Data_Sheet_1.DOC]

**Appendix:** Stimuli List for Experiment 2.

|  | Stimuli | Stroke | Familiarity | Phonological similarity to Chinese |
| --- | --- | --- | --- | --- |
| Sino-Korean* | 신문^1^ | 13 | 4.5 | 6.3 |
|  | 부장^2^ | 12 | 3.9 | 4.4 |
|  | 은행^3^ | 11 | 5.4 | 5.6 |
|  | 교통^4^ | 12 | 4.6 | 4.8 |
|  | 농민^5^ | 10 | 3.7 | 5.7 |
|  | 항공^6^ | 11 | 5.3 | 5.2 |
|  | 부문^7^ | 14 | 3.7 | 4.7 |
|  | 생일^8^ | 13 | 6.1 | 4.6 |
| Pure words | 분야 | 12 | 3.2 | 2.3 |
|  | 가방 | 11 | 5.5 | 1.5 |
|  | 나무 | 10 | 5.7 | 2.1 |
|  | 가을 | 11 | 4.7 | 1.3 |
|  | 날씨 | 14 | 4.6 | 1.8 |
|  | 여가 | 8 | 3.1 | 1.4 |
|  | 가만 | 12 | 2.7 | 2.6 |
|  | 지금 | 11 | 6.2 | 1.3 |
| Korean dissyllabic non-word * | 씨당^9^ | 11 |  | 6.1 |
|  | 신리^10^ | 11 |  | 4.5 |
|  | 구향^11^ | 11 |  | 5.5 |
|  | 통심^12^ | 14 |  | 5.3 |
|  | 심용^13^ | 12 |  | 5.5 |
|  | 푸통^14^ | 13 |  | 5.1 |
|  | 다회^15^ | 11 |  | 5.1 |
|  | 회이^16^ | 8 |  | 6.2 |
| Pure non-word | 분아 | 11 |  | 1.3 |
|  | 가벙 | 11 |  | 1.6 |
|  | 뇨무 | 11 |  | 1.6 |
|  | 가읈 | 12 |  | 2 |
|  | 낱씨 | 13 |  | 1.5 |
|  | 왜가 | 10 |  | 1.6 |
|  | 게만 | 13 |  | 1.4 |
|  | 리금 | 13 |  | 1.8 |

*Note*. Sino-Korean = W+C+; Pure words = W+C-; Korean syllables = W-C+; Pure non-word = W-C-

*

| Chinese origin | Frequency count |
| --- | --- |
| 1新闻 | 671 |
| 2部长 | 729 |
| 3银行 | 449 |
| 4交通 | 293 |
| 5农民 | 1320 |
| 6航空 | 138 |
| 7部门 | 506 |
| 8生日 | 526 |

| Chinese origin | Frequency count |
| --- | --- |
| 9食堂 | 398 |
| 10心理 | 1132 |
| 11故乡 | 402 |
| 12通信 | 211 |
| 13信用 | 127 |
| 14普通 | 882 |
| 15大会 | 562 |
| 16会议 | 883 |
